# 0Effects of the COVID-19 Pandemic on Treatment Adherence in Patients with Chronic Heart Failure

**DOI:** 10.22088/cjim.13.0.199

**Published:** 2022

**Authors:** Sergey Yu. Martsevich, Yulia V. Lukina, Natalia P. Kutishenko, Elmira T. Guseynova

**Affiliations:** 1 Department of Preventive Pharmacotherapy, National Medical Research Center for Therapy and Preventive Medicine, Petroverigsky per. 10, Moscow, Russia

**Keywords:** COVID-19 pandemic, chronic heart failure, period of home isolation, adherence to treatment, factors of non-adherence

## Abstract

**Background:**

To assess the influence of the COVID-19 (Coronavirus Disease 2019**)** pandemic on treatment adherence by patients with CHF (Chronic heart failure) and to determine the factors associated with changing adherence during home-isolation.

**Methods:**

The survey was conducted in patients participating in the COMPLIANCE study (ClinicalTrials.gov. NCT04262583). Thirty-one patients, included into in the COMPLIANCE study before March 1, 2020, were interviewed through phone calls. A modified adherence scale of the National Society for Evidence-Based Pharmacotherapy was used, which permits to assess of overall adherence, adherence to particular drugs and the main causes for non-adherence.

**Results:**

In the whole group of patients, only a tendency to reduced overall adherence was registered during the COVID-19 pandemic (р=0,256). Significant differences in the rate of adherence deterioration were revealed for angiotensin-converting enzyme (ACE) inhibitors (p=0.031) and for statins (p=0.002). The reasons for non-adherence were lack of opportunity to contact with a physician, inability to perform investigations necessary for correcting prescribed pharmacotherapy, and side effects.

**Conclusion:**

A tendency to deterioration of adherence to prescribed pharmacotherapy was revealed during the COVID-19 pandemic. A significant decline in adherence was registered to ACE inhibitors and statins.

On March 11, 2020, the World Health Organization announced the COVID-19 (Corona VIrus Disease 2019) pandemic. This has led to changes in the work of health systems in many countries of the world. Governments of several countries have implemented strict stay-at-home policies in connection with the pandemic. This period has limited patients' access to medical care (1). Also, various problems of pharmacotherapy in these patients have become aggravated, i.e. its availability, safety, drug-drug interactions with additional prescriptions, etc. (2-4). In the absence of constant patient-doctor contact during the home-isolation period, the problem of adherence of patients with chronic non-communicable diseases (CNCDs) to treatment became even more significant. Various changes in pharmacotherapy or complete withdrawal of medications during an unfavorable epidemiological period may worsen the course of CNCDs and increase the risk of complications (1, 5, 6). The purpose of this article is to assess the adherence of patients with CHF to recommended medication during the period of home-isolation in the COVID-19 pandemic, to compare it with pre-pandemic rates and to determine the main factors associated with changing adherence.

## Methods

The survey was conducted on patients participating in the COMPLIANCE study (Clinical Trials.gov. NCT04262583). Patients from the outpatient registry of the specialized cardiology department of the medical center are included in this study (7). Since the course of the COMPLIANCE study coincided with the COVID-19 pandemic, it was decided to perform an additional survey to assess the influence of the COVID-19 pandemic on patients’ adherence to previously prescribed drug therapy. Among 80 patients included in the study, 31 were interviewed on adherence between 11 and 25 May 2020 (mean age: 66±11 years; 23 men, 8 women). We were not able to interview all the patients due to the self-isolation regime and the doctors’ inability to contact all patients. In a telephone survey, we assessed patients' adherence to the recommended pharmacotherapy and its dynamics during the period of home-isolation. We also determined the main factors associated with changing adherence during the home-isolation. A modified National Society for Evidence-based Pharmacotherapy (NODPh) adherence scale was used to assess adherence. The scale was supplemented with questions about the reasons for non-adherence during the COVID-19 pandemic. 

The scale of adherence to NODPh allows defining both overall adherence to pharmacotherapy, and adherence to each drug separately. Patients who adhered to all medical guidelines were rated as completely adherent (“0” on the NODF Scale), those intentionally or unintentionally violating a drug regime were considered partially adherent (“1-2” on the NODPh Scale), and those who stopped taking one, several or all drugs were regarded as non-adherent (3). In dichotomous division, subgroups of complete adherent patients and patients with disorders of adherence to pharmacotherapy were distinguished. Adherence to specific drugs was also assessed on the modified National Society for Evidence-based Pharmacotherapy (NODPh) adherence scale (8). All COMPLIANCE study documents, including the modified version of the scale of the NODPh adherence scale with additional questions, were approved by an Independent Ethics Committee. All patients gave informed consent to include personal data in the registry and to participate in the COMPLIANCE study and surveys related to it. The additional survey is represented in the link 1. All questions in the questionnaire were closed-ended. We compared patient adherence before the pandemic (after inclusion in the COMPLIANCE study) with that of during the pandemic. In addition, a comparison was made in two subgroups of patients: new and established patients [9]. New patients were enrolled in the outpatient registry and the COMPLIANCE study simultaneously, during V1. The established patients were included in the registry prior to the inclusion visit in the observational study and were observed specifically**.**

The statistical analysis was performed using IBM SPSS Statistics 23.0 software (IBSM, USA). The main descriptive statistics for categorical and ordinal variables were the frequency and proportion (%); mean, standard deviation, minimum (min.), and maximum (max.) or median, and interquartile range for quantitative variables. For comparative analysis the χ2 test, Fisher's exact test, z-test, McNemar's test (Yates correction for continuity) were used. 

## Results

**Figure 1:**
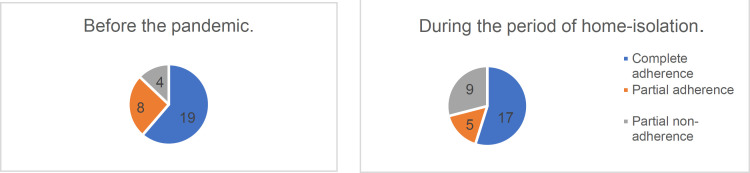
Dynamics of overall treatment adherence (NODPh adherence scale) Dynamics of overall treatment adherence before and during the pandemic COVID-19 (n=31)

The clinical and demographic characteristics of patients (n=31) who were interviewed by telephone during the home isolation period are presented in Table 1 and 2. Out of 31 patients, 18 were under medical supervision in the medical center for a long time (since 2016), and 13 turned to help to the medical center for the first time (since 2019). The results of the telephone survey showed that the number of complete and partially adherent patients decreased, while the number of non-adherent patients doubled (figure 1). 

However, the revealed differences did not reach statistical significance (p=0.256). The dynamics of adherence to specific drugs is shown in Table 2. In a telephone survey, we recorded cases of drug discontinuation of various patients with CHF. The most pronounced decline in adherence was revealed for ACE inhibitors (p=0.031) and for statins (p=0.081). The difference in adherence changes was revealed between the new patients and established patients who were under medical supervision in the medical center previously. A decline in overall adherence to pharmacotherapy was recorded in 61.5% of new patients and in 16.7% of established patients (p=0.01); OR=8.0 DI 95% [1.5; 42.5], p=0.02.

**Table 1:** Clinical and demographic characteristics of patients (n=31)

**Characteristics **	**n, %**
Mean age ± SD, years	66.6±10,8
Males	23 (74.2%)
HF (NYHA class)	
Class -I	6 (19.4%)
Class -II	19 (61.2%)
Class -III	6 (19.4%)
EF≤40%	5 (16.1%)
EF>40%	26 (83.9%)
CAD	25 (80.6%)
MI	20 (64.5%)
Arterial hypertension	25 (80.6%)
Atrial fibrillation	15 (48.4%)
Diabetes mellitus	11 (35.5%)

Notes: SD - standard deviation, HF- Heart failure; EF- ejection fraction; CAD- Chronic coronary artery disease, MI- myocardial infarction; NYHA- New York Heart Association;

**Table 2:** Comparative characteristics of adherence between new and established patients

		**new patients ** **n=13**		**established supervised n=18**		p
		**n**	**% **	**n**	**% **	
Mean age ±SD, years		68.1±11.2		65.7±11.5		
HF (NYHA class)	1	2	15.4%	4	22.2%	0.84
	2	8	61.5%	11	61.1%	
	3	3	23.1%	3	16.7%	
Atrial fibrillation		8	61.5%	7	38.9%	0.21
MI	1	6	46.2%	14	77.8%	0.07
COPD	1	5	38.5%	5	27.8%	0.53
Diabetes mellitus	1	3	23.1%	8	44.4%	0.22
CAD (Grading of angina)	0	5	38.5%	1	5.6%	<0.022
	1	2	15.4%	5	27.8%	0.14
	2	4	30.8%	9	50.0%	
	3	2	15.4%	3	16.7%	

Notes: EF- ejection fraction; HF- Heart failure; MI- myocardial infarction; NYHA- New York Heart Association; SD - standard deviation. CAD - Chronic coronary artery disease. Also, statistically significant differences in adherence to ACE inhibitors (p=0.008) and statins (p=0.002) were found between new patients and established patients. All established patients (100%) remained adherent to statins. ACE inhibitors were taken in strict compliance with medical recommendations by 88.2% of those who were monitored for a long time at the medical center. Violations of adherence to ACE inhibitors and statins were found in 60% of new patients during the pandemic (figure 2). 

**Figure 2:**
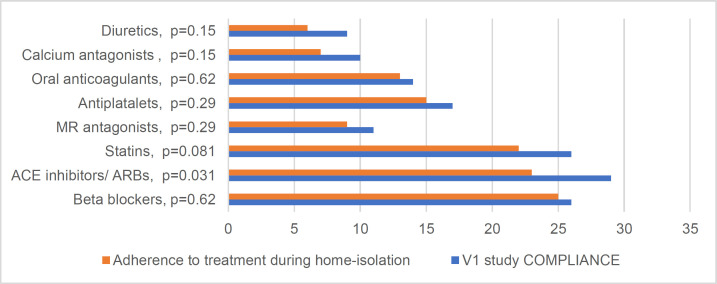
Dynamics of adherence to specific drugs

The reasons for the violation’s overall adherence and the adherence to the specific drugs were: the inability to contact a physician to clarify questions on pharmacotherapy, and the inability to take tests to correct the dose of drugs. The leading reasons for the lack of adherence to ACE inhibitors were side effects (excessive reduction in blood pressure) and the inability to consult a physician on the necessary correction of the therapy.

## Discussion

The New Coronavirus Infection Pandemic demonstrated the continuing relevance of the problem of adherence to pharmacotherapy for patients with CNCD. It is known that patients may have different adherence to different drugs. According to ST de Vries et al, different adherence to hypolipidemic, antihypertensive and oral antidiabetic drugs has been identified in patients with CHF (10). 

According to the results of the telephone survey of CHF patients, we revealed a tendency in the overall adherence decline. Analysis of adherence to specific classes of drugs showed different results. There was a statistically significant deterioration of adherence for ACE inhibitors and statins. This data differs from the results of a study by M.Viana et al. according to which patients with CHF (outside of adverse epidemiological conditions) have the highest adherence rates to ACE inhibitors (11). These drugs have a high significance and positive effects on the life prognosis in CHF patients. Therefore, cessation of ACE inhibitors therapy during COVID-19 pandemic may be potentially dangerous. 

Since the first questionnaire of patients with chronic stable coronary artery disease (SCAD) during COVID-19 pandemic (in press) did not permit us to assess of them due to specific reasons for non-adherence, we made several changes to the adherence questionnaire. For patients with CHF the questionnaire was supplemented with questions about the main barriers to adherence during the period of home-isolation. The identified reasons showed overall lack of adherence and the factors that led CHF patients to stop taking specific drugs. 

The main reasons to discontinue ACE were side effects and the inability to obtain clarifying information about pharmacotherapy from the physician during the period of home-isolation. This was true mainly for primary patients of COMPLIENCE study. These patients can be assured of being unaware of these drugs role to improve outcomes of CHF. 

The results obtained in our study were compared with data from other studies to assess the adherence of patients with different CNCD during the period of home-isolation. It should be mentioned that these studies are inconsistent and the data is heterogeneous. Khan N et al. during the COVID-19 pandemic revealed an 11% decline in the adherence rate of patients with inflammatory bowel diseases to infusion biological therapy compared to the pre-home-isolation period (7). These data were not confirmed in another similar paper by Barberio B. et al, which showed that patient’s adherence during the pandemic was maintained at a high level. This was achieved by the usage of telemedical technology (13). Deterioration of adherence during the pandemic was confirmed in psoriasis patients AND in patients with rheumatic inflammatory diseases, according to telephone surveys (5, 6). In contrast, a study by Kostev K et al. of the IMS long-term prescription database showed an increase in the number of patients who took a recipe for medicine in March 2020 compared to the same period last year (14). 

Increased adherence to treatment during the COVID-19 pandemic was also identified in a study by Kaye L. et al. based on the analysis of electronic medication data usage and digital platform that tracks inhaler usage through electronic medication monitors; it was shown that adherence in patients with bronchial asthma and chronic obstructive pulmonary disease (COPD) increased by 14% on average. A possible reason for this is the high motivation of patients with respiratory system disorders to follow medical guidelines during the COVID-19 pandemic. According to the authors, the results obtained cannot be transferred to patients with other chronic diseases (15). The problem of adherence to medication during the COVID-19 pandemic needs further study. Maintaining patient-doctor contact, informing patients about the importance of following medical recommendations and using telemedicine technologies during this difficult epidemiological period seems to be very important to achieve these objectives. 

**Limitations of the study:** This was a single-center study that investigated the involvement of a small number of patients. There was the difficulty of distinguishing between factors clearly related to the period of home-isolation during COVID-19 pandemic, and those that were unrelated to this period.

In conclusion during the home-isolation period of the COVID-19 pandemic, there was a decline in drug adherence in patients with CHF. This is likely due to the difficulty or impossibility of patients to get in touch with their treating physician. The most pronounced decrease in the rate of adherence was registered for ACE inhibitors and statins. The highest risk of adherence violations during the period of self-isolation was observed in new patients who had not been previously supervised in one of the medical centers.
